# General synthesis of hierarchical sheet/plate-like M-BDC (M = Cu, Mn, Ni, and Zr) metal–organic frameworks for electrochemical non-enzymatic glucose sensing†

**DOI:** 10.1039/c9sc05636j

**Published:** 2020-03-12

**Authors:** Gilang Gumilar, Yusuf Valentino Kaneti, Joel Henzie, Sauvik Chatterjee, Jongbeom Na, Brian Yuliarto, Nugraha Nugraha, Aep Patah, Asim Bhaumik, Yusuke Yamauchi

**Affiliations:** Welding and Fabrication Engineering Technology Department, Institut Teknologi Sains Bandung Central Cikarang Bekasi 17530 Indonesia; Advanced Functional Materials (AFM) Laboratory, Engineering Physics Department, Institut Teknologi Bandung Bandung 40132 Indonesia brian@tf.itb.ac.id; International Center for Materials Nanoarchitectonics (WPI-MANA), National Institute for Materials Science (NIMS) 1-1 Namiki Tsukuba Ibaraki 305-0044 Japan KANETI.Valentino@nims.go.jp; School of Materials Sciences, Indian Association for the Cultivation of Science Jadavpur Kolkata 700-032 India; School of Chemical Engineering, Australian Institute for Bioengineering and Nanotechnology (AIBN), The University of Queensland Brisbane QLD 4072 Australia y.yamauchi@uq.edu.au; Research Center for Nanoscience and Nanotechnology (RCNN), Institut Teknologi Bandung Bandung 40132 Indonesia; Inorganic & Physical Chemistry Research Division, Institut Teknologi Bandung Bandung 40132 Indonesia; Department of Plant and Environmental New Resources, Kyung Hee University 1732 Deogyeong-daero, Giheung-gu Yongin-si Gyeonggi-do 446-701 South Korea

## Abstract

Two-dimensional metal–organic frameworks (2D MOFs) are an attractive platform to develop new kinds of catalysts because of their structural tunability and large specific surface area that exposes numerous active sites. In this work, we report a general method to synthesize benzene dicarboxylic acid (BDC)-based MOFs with hierarchical 3D morphologies composed of 2D nanosheets or nanoplates. In our proposed strategy, acetonitrile helps solvate the metal ions in solution and affects the morphology, while polyvinylpyrrolidone (PVP) serves as a shape-control agent to assist in the nucleation and growth of MOF nanosheets. PVP also acts as a depletion agent to drive the assembly of the hierarchical sheet/plate-like M-BDC under solvothermal conditions. Further, we also demonstrate the flexibility of the proposed method using numerous coordinating metal ions (M = Cu, Mn, Ni, and Zr). The potential of these MOFs for electrochemical glucose sensing is examined using the hierarchical sheet-like Ni-BDC MOF as the optimum sample. It drives the electrocatalytic oxidation of glucose over a wide range (0.01 mM to 0.8 mM) with high sensitivity (635.9 μA mM^−1^ cm^−2^) in the absence of modification with carbon or the use of conductive substrates. It also demonstrates good selectivity with low limit of detection (LoD = 6.68 μM; signal/noise = 3), and fast response time (<5 s).

## Abstract 2

This work reports the general fabrication of hierarchical sheet/plate-like M-BDC MOFs for electrochemical non-enzymatic glucose sensing.

## Graphical abstract

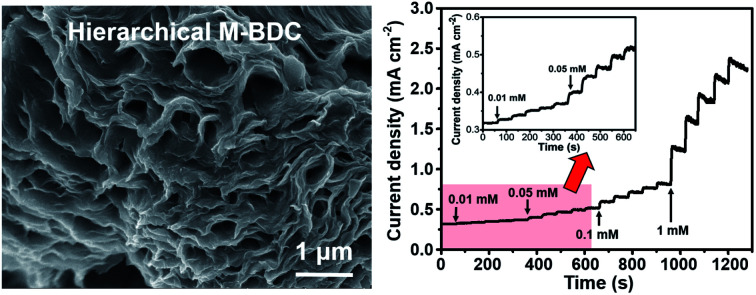

## Introduction

Metal–organic frameworks (MOFs) are porous crystalline materials formed by the coordination of metal ions with organic linkers.^1–4^ The metal clusters in MOFs act as joints, and the organic linkers act as struts to form an interconnected network *via* coordination bonds and intermolecular interactions.^5^ The tunable pore geometries and flexible frameworks of MOFs have been exploited to develop new kinds of ultrahigh surface area materials for gas storage and adsorption, while the metal centers in MOFs have been examined as active sites to drive catalytic reactions.^6–14^ The combination of high surface area and catalytic activity of MOF architectures is promising as a platform in sensing applications, in part due to the numerous kinds of bonding interactions available to the analyte.^6,15,16^ However despite these favorable attributes, the bulk three-dimensional (3D) morphologies of most MOFs (*e.g.*, octahedra, dodecahedra, or polyhedra) limit mass or ion transport and reduce access to the active sites.^17–19^ Therefore, developing synthetic methods that enable effective morphological tuning of MOFs while still maintaining their catalytic properties and high surface area is desirable.

Two-dimensional (2D) MOFs have unique physical and chemical properties that arise from electronic effects caused by their small thicknesses, large surface areas, and high surface-to-volume atom ratios.^20^ 2D MOFs have shown superior performance compared to bulk 3D MOFs in numerous applications. For example, 2D MOFs showed dramatically improved dye adsorption compared to 3D MOFs due to the improved diffusion and better access to interior Lewis acid sites.^19,21^ In addition, the ease of access to the active sites of 2D MOFs could promote enhanced interactions between the active sites and target molecules in sensing applications, leading to high sensitivity.^22^ 2D MOFs have been synthesized using both top-down and bottom-up strategies. Top-down strategies typically include delamination, mechanical exfoliation, sonication exfoliation, and chemical exfoliation.^23–25^ Although these strategies can produce high quality single- or few-layer nanosheets, they are usually time-consuming and suffer from low yield.^26^ In comparison, the bottom-up approaches including interfacial synthesis, three-layer synthesis, surfactant-assisted synthesis, modulated synthesis, and sonication synthesis are more straightforward and generate larger yields but are challenging to control the morphology precisely.^27–29^

Ultrathin 2D nanosheets can support enhanced electrocatalytic activity, but they tend to be less stable than 3D structures because they are prone to restacking. In this regard, the construction of hierarchical 3D MOF nanostructures is attractive because it may be engineered to maintain many of the aspects of 2D MOFs (*i.e.*, large surface area, interconnected open pores, rich redox sites) but in a hierarchical framework that promotes stability for electrochemical applications. Previously, a hierarchical flower-like Ni-MOF [Ni_3_(OH)_2_(PTA)_2_(H_2_O)_4_]·2H_2_O (PTA = *p*-benzenedicarboxylic acid) was prepared by the hydrothermal reaction between Ni^2+^ and PTA at 150 °C. The resulting Ni-MOF showed high electrocatalytic activity and good stability for glucose oxidation reaction.^30^ In addition, hierarchical ZIF nest architectures were previously synthesized by the solvothermal reaction of zinc nitrate hexahydrate with 2-ethylimidazole and 5,6-dimethylbenzimidazole in a mixed solvent of methanol and aqueous ammonia.^31^ Despite these achievements, it is still challenging to develop a generalized route for fabricating hierarchical 3D MOFs assembled from 2D nanostructures, such as nanosheets/nanoplates.

Glucose sensing plays an essential role in the detection of diabetes. Enzymatic sensors display high sensitivity and selectivity toward glucose. However, natural enzymes suffer from some disadvantages, such as high cost, poor long-term stability, and complex immobilization process. Alternative glucose detection methods are being developed using sensors that employ light and acoustic waves, in addition to electrochemical methods. Among them, electrochemical glucose sensors are particularly attractive due to their simplicity, low cost, high sensitivity, and rapid response. Noble metals and their alloys are frequently utilized for electrochemical non-enzymatic glucose sensing owing to their high response and excellent selectivity. However, the expensive nature of these materials has impeded their large-scale applications. Therefore, there is a need to develop low-cost and high-performance catalysts for non-enzymatic glucose sensing. The direct utilization of MOFs as electrochemical glucose sensors is still limited due to the low electrical conductivity of pristine MOFs. Previously, Cu MOF-modified electrode showed a relatively good electrocatalytic activity for glucose oxidation in the linear range of 0.06 μM to 5 mM with a sensitivity of 89 μA mM^−1^ cm^2^ and a detection limit of 10.5 nM.^32^ In another report, spheroidal Ni-MOF particles showed poor performance for electrochemical glucose sensing when utilized alone. However, their hybridization with carbon nanotubes (CNTs) yielded a higher sensitivity of 13.85 mA mM^−1^ cm^−2^, a low detection limit of 0.82 μm, and a wide linear range of 1 μM to 1.6 mM.^33^ Despite some success in preparing pure MOF electrodes, electrochemical glucose sensing with them is challenging because they have low sensitivity. Therefore, it is essential to create a hierarchical MOF architecture that enables control of size, structure, and composition.

In this work, we report the general synthesis of hierarchical sheet/plate-like M-BDC nanosheets, where M = Cu, Mn, Ni, and Zr, and BDC = benzene-1,4-dicarboxylic acid. These MOFs are prepared in a solvothermal reaction by combining different metal nitrate precursors with the BDC ligand in the presence of polyvinylpyrrolidone (PVP) and acetonitrile as shape-directing agents. This polymer–solvent system promotes good solvation of the metal ions and leads to the formation of hierarchical sheet/plate-like M-BDC MOFs. When evaluated for electrochemical glucose sensing, the as-prepared hierarchical sheet-like Ni-BDC electrode exhibits superior electrocatalytic oxidation toward glucose in the range of 0.01 mM to 0.8 mM with a relatively high sensitivity of 635.9 μA mM^−1^ cm^−2^, without any modification with carbon or the use of a conductive substrate. Furthermore, the hierarchical sheet-like Ni-BDC sensor has a relatively good selectivity with a low limit of detection (LoD) of 6.68 μM (signal/noise = 3), and fast response time (<5 s).

## 2. Experimental procedures

### 2.1. Chemicals

Copper(ii) nitrate trihydrate (Cu(NO_3_)_2_·3H_2_O, 99.5%), nickel(ii) nitrate hexahydrate (Ni(NO_3_)_2_·6H_2_O, 99.5%), manganese(ii) nitrate tetrahydrate (Mn(NO_3_)_2_·4H_2_O, 99.5%), d(+)-glucose (C_6_H_12_O_6_, 99.5%), *N*,*N*-dimethylformamide (DMF), and acetonitrile (C_2_H_3_N, 99.8%) were purchased from Fujifilm Wako Pure Chemical Corporation (Japan). Benzene-1,4-dicarboxylic acid or terephthalic acid (H_2_BDC, 98%), uric acid (C_5_H_4_N_4_O_3_, ≥99%), and zirconium(iv) chloride (ZrCl_4_, 99%) were purchased from Sigma-Aldrich (Japan). Polyvinylpyrrolidone K-30 (PVP) (*M*_w_ = 40 000), maltose (C_12_H_22_O_11_, ≥99%), and l-ascorbic acid (C_6_H_8_O_6_, 99%) were purchased from Nacalai Tesque (Japan).

### 2.2. General synthesis of hierarchical sheet/plate-like M-BDC (M = Cu, Mn, Ni, and Zr)

In a typical procedure, 0.3 g of the metal nitrate precursor was dissolved in a solvent mixture containing 10 mL of DMF and 30 mL of acetonitrile to form solution A. In a separate bottle, 0.3 g of H_2_BDC was dissolved in a mixture containing 30 mL of DMF and 10 mL of acetonitrile, followed by the addition of PVP into the mixture solution under stirring to form solution B. The optimized mass ratios of metal precursor to PVP are 1 : 3 for Cu-BDC and Ni-BDC, and 1 : 5 for Mn-BDC and Zr-BDC (*i.e.*, 0.9 g of PVP was used to prepare Cu-BDC and Ni-BDC and 1.5 g of PVP was used to synthesize Mn-BDC and Zr-BDC). Next, 4 mL of solution A was mixed with 4 mL of solution B in a 50 mL vial, and then the mixture was sonicated for 2 min. The mixed solution was subsequently heated in an oil bath at 135 °C for 24 h without stirring. The resulting precipitates were centrifuged at 14 000 rpm for 8 min, washed consecutively with DMF and methanol for three times each and then dried in air at 60 °C.

### 2.3. Characterization

The morphological characterization of the MOF samples was performed with a scanning electron microscope (SEM, Hitachi SU-8000) operated at an accelerating voltage of 10 kV. The composition and crystal structure of the MOF products were checked by X-ray diffraction (XRD, Rigaku RINT 2500X) with Cu-Kα radiation (*λ* = 0.15406 nm). Rietveld refinement analysis was carried out using EXPO2014 software.^34^ The Fourier transform infrared (FTIR) spectra of the samples were collected by using a Thermo Scientific Nicolet 4700 in the wavenumber region of 500 to 4000 cm^−1^. Nitrogen (N_2_) adsorption–desorption measurements of the MOF samples were carried out by using BELSORP-max (BEL, Japan) at 77 K. The specific surface area and pore size distribution of the samples were calculated by employing Brunauer–Emmett–Teller (BET) and nonlocal density functional theory (NLDFT) methods, respectively. Before the BET measurement, each sample was degassed at 175 °C for 20 h to remove adsorbed moisture. The post-cycling measurement of nickel concentration in the glucose-containing NaOH solution was performed by atomic absorption spectroscopy using GBC SavantAA atomic absorption spectrophotometer (GBC Scientific Equipment, Australia) at a wavelength of 232 nm.

### 2.4. Non-enzymatic glucose sensing measurements

The electrochemical measurements were performed with an electrochemical workstation (CHI-660, USA) using a three-electrode system consisting of glassy carbon electrode (GCE) as the working electrode, platinum wire as the counter electrode, and Ag/AgCl as the reference electrode. The working electrode was prepared by drop-casting 5 μL of the MOF suspension onto a clean and polished glassy carbon electrode (GCE). The suspension was prepared by dispersing 5 mg of the MOF powder in 900 μL of isopropanol, followed by the addition of 100 μL of Nafion and subsequent sonication for 30 minutes. The electrolyte used was 0.1 M NaOH, and the glucose solutions were dissolved in 0.1 M NaOH at various concentrations (0.1–20 mM). The stability test was carried out by measuring the variation of the current response of the hierarchical sheet-like Ni-BDC electrode to 0.1 mM glucose for 6 consecutive cycles after 50 days of storage time.

## 3. Results and discussion

### 3.1. Synthesis and characterization of hierarchical sheet/plate-like M-BDC MOFs

**Fig. 1:**
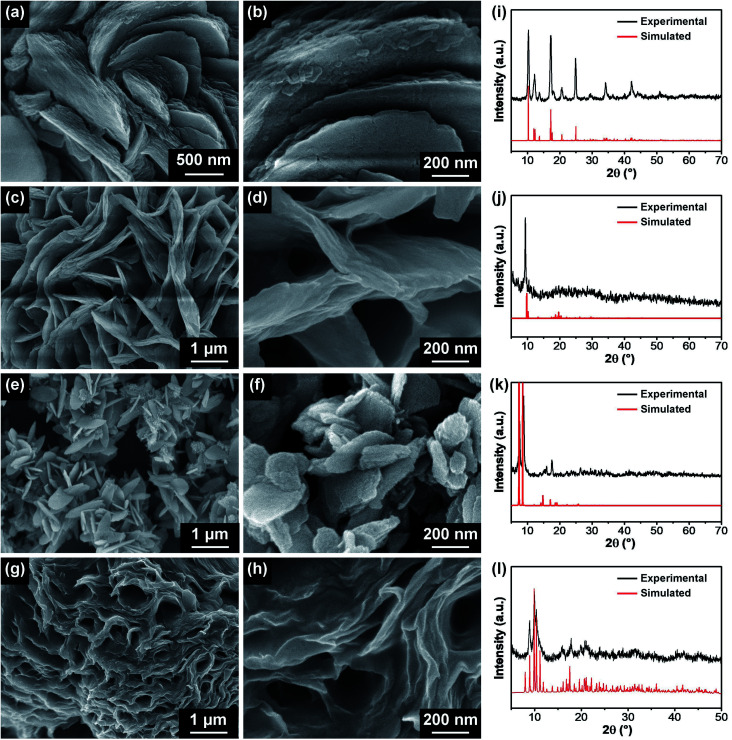
Low- and high-magnification SEM images of M-BDC products obtained with the optimized mass ratios of metal precursor to PVP: (a and b) Cu-BDC (1 : 3), (c and d) Mn-BDC (1 : 5), (e and f) Zr-BDC (1 : 5) and (g and h) Ni-BDC (1 : 3). Experimental, simulated and reference PXRD patterns for Cu-BDC(i), Mn-BDC (j), and Zr-BDC (k). (l) Experimental and simulated PXRD patterns for Ni-BDC.

The synthesis of the hierarchical sheet/plate-like M-BDC particles was performed in an oil bath at 135 °C in the presence of both PVP and acetonitrile as structure-directing agents. The general synthetic procedure works for several different metals, but each type of M-BDC requires a different mass ratio of metal to PVP to achieve the hierarchical sheet/plate-like morphology (*i.e.*, M : PVP = 1 : 3 for Cu-BDC and Ni-BDC and 1 : 5 for Mn-BDC and Zr-BDC). SEM images of the optimized M-BDC MOFs are given in Fig. 1. The Cu-BDC sample displays a hierarchical architecture formed by the stacking of several square-like plates (Fig. 1a and b). In comparison, the optimized Mn-BDC product shows a hierarchical flower-like morphology which is assembled from nanoplates (Fig. 1c and d), whereas the optimized Zr-BDC sample has hierarchical plate-like morphology with lengths of ∼800 nm to 1 μm (Fig. 1e and f). In contrast, the optimized Ni-BDC product exhibits a hierarchical multilayered sheet-like structure (Fig. 1g and h).

The hierarchical plate-like Cu-BDC exhibits major peaks at around 10.3°, 12.2°, 13.6°, 17.1°, 18.0°, 20.6°, 24.8°, 34.1°, and 42.1°, assigned to (110), (020), (111̄), (201̄), (111), (220), (4̄02), and (242) planes of Cu-BDC, respectively (CCDC no. 687690) (Fig. 1i).^28^ Rietveld refinement was carried out on the powder XRD (PXRD) pattern of the Cu-BDC product and the refined unit cell parameters are identified to be *a* = 11.32 Å, *b* = 14.33 Å, and *c* = 7.78 Å. As shown in Fig. 1j, the Mn-BDC product exhibits two major peaks at around 9.86° and 10.4°, indexed to (111̄) and (202̄) planes of Mn-BDC, respectively (CCDC no. 265094), which is in good agreement with the report of Rosi *et al.*^35^ The refined unit cell parameters for Mn-BDC are *a* = 24.79 Å, *b* = 10.59 Å, and *c* = 17.42 Å. The as-prepared Zr-BDC (more commonly known as UiO-66) sample displays strong peaks at around 7.2°, 8.8°, and 17.4° (Fig. 1k) which match well with the (111), (200), and (400) planes of Zr-BDC (UiO-66), respectively (CCDC no. 733458), in agreement with the work of Cavka *et al.*^36^ The refined unit cell parameters for Zr-BDC are *a* = 24.79 Å, *b* = 14.33 Å, and *c* = 7.78 Å. The indexing of the powder XRD pattern of Ni-BDC (Fig. 1l) was performed using EXPO2014 software for generating the lattice parameters.^34^ Rietveld refinement was performed based on a model which gives a closely resembled XRD pattern for Ni-BDC. The refined PXRD pattern can be assigned to monoclinic phase with space group *P*12_1_/*n*1 with unit cell parameters: *a* = 12.98 Å, *b* = 11.38 Å, and *c* = 17.90 Å (*α* = *β* = 90° and *γ* = 96.7°). The details of the refined unit cell parameters of these M-BDC MOFs are given in Table S1.†

**Fig. 2:**
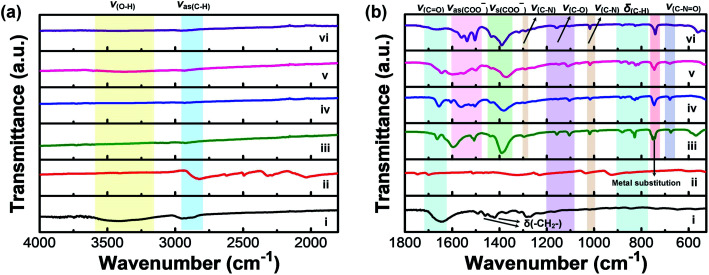
FTIR spectra of (i) pure PVP, (ii) H_2_BDC ligand and the as-prepared M-BDC products: (iii) Cu-BDC, (iv) Mn-BDC, (v) Ni-BDC, and (vi) Zr-BDC in the wavenumber regions of (a) 4000–1800 cm^−1^ and (b) 1800–525 cm^−1^.

FTIR measurements were carried out to further confirm the formation of M-BDC MOFs (Fig. 2). Fig. 2a and b display the FTIR spectra of pure PVP, the BDC ligand (terephthalic acid), and the as-synthesized M-BDC MOFs in the wavenumber regions of 4000–1800 cm^−1^ and 1800–525 cm^−1^, respectively. The FTIR spectrum of the pure PVP shows a broad peak between 3200–3600 cm^−1^, which can be assigned to the stretching vibration of O–H (Fig. 2a(i)). The IR bands between 2850–2950 cm^−1^ can be indexed to the asymmetric stretching vibration of CH_2_ in the skeletal chain of PVP. The vibration band of C=O group is observed at 1642 cm^−1^ (Fig. 2b(i)), indicating the presence of carbonyl groups in PVP.^37^ The peaks at 1462 cm^−1^ and 1371 cm^−1^ are assignable to the bending vibration of CH_2_, whereas the peak at around 1440 cm^−1^ is indexable to the bending vibration of O–H. The peak located between 1285–1295 cm^−1^ matches the stretching vibration of C–N in PVP. The deprotonation of H_2_BDC is confirmed by the shift of the C=O stretching vibration at 1700 cm^−1^ (Fig. 2b(ii)) to ∼1665 cm^−1^ for the M-BDC MOFs (Fig. 2b(iii)–(vi)). Furthermore, the M-BDC MOFs display sharp peaks in the regions of 1490–1600 cm^−1^ and 1350–1450 cm^−1^, which match the asymmetric and symmetric stretching vibrations of the carboxyl group, respectively.^33^ The separation between these two modes indicates that the COO^−^ of BDC ligand is coordinated to the metals through a bidentate mode.^38^ The IR bands between 1080–1200 cm^−1^ in the FTIR spectra of M-BDC MOFs can be assigned to the C–O stretching vibration. The presence of the C–N stretching of aromatic amines is observed between 1293–1300 cm^−1^, whereas the C–N stretching of aliphatic amines is located at around 1019 cm^−1^. The bending vibration of C–N=O appears at around 675 cm^−1^ for M-BDC MOFs.^39^ In addition, the IR bands between 750–880 cm^−1^ can be assigned to the C–H bending vibration. The IR band located at 740 cm^−1^ can be indexed to the metal substitution on benzene groups.^40^ The strong bands at around 540 and 670 cm^−1^ are assigned to O–M(metal)–O vibrations. The presence of these new IR bands indicates the successful coordination of the metals with BDC ligands to form M-BDC MOFs. These FTIR results further confirm the successful formation of M-BDC MOFs.

**Fig. 3:**
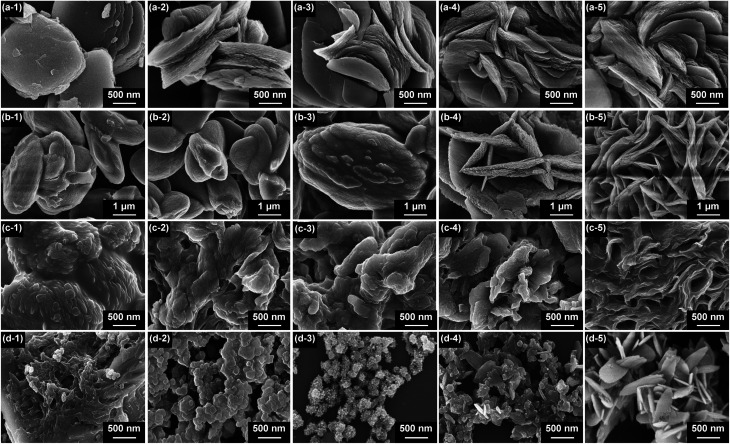
SEM images showing the morphological evolutions of (a) Cu-BDC, (b) Mn-BDC, (c) Ni-BDC, and (d) Zr-BDC with increasing reaction time: (1) 2 h; (2) 4 h; (3) 8 h; (4) 16 h, and (5) 24 h.

**Fig. 4:**
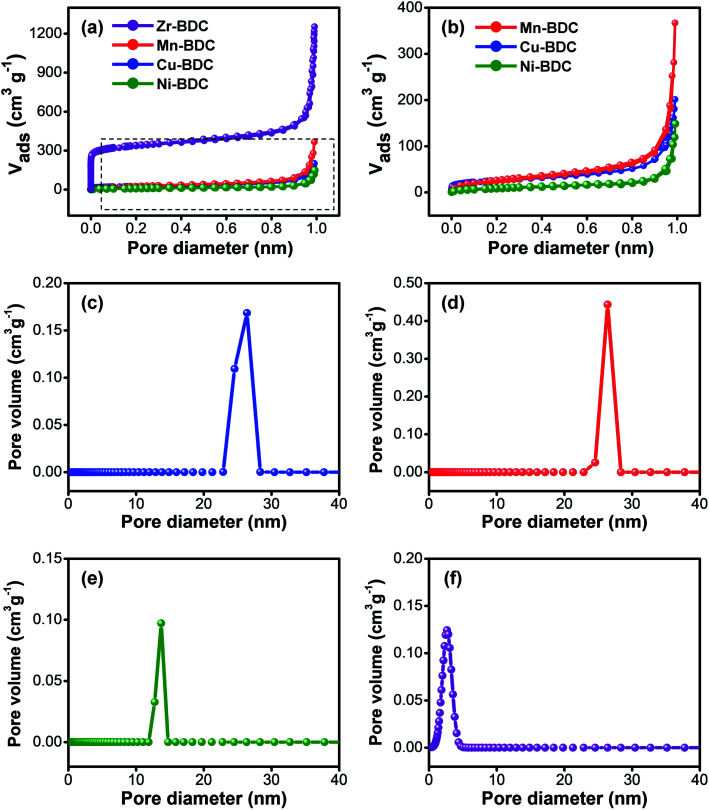
(a) Nitrogen (N_2_) sorption isotherms of all prepared M-BDC products. (b) Enlarged N_2_ sorption isotherms of the as-synthesized Mn-BDC, Cu-BDC, and Ni-BDC samples. NLDFT pore size distribution plots of (c) Cu-BDC, (d) Mn-BDC, (e) Ni-BDC, and (f) Zr-BDC.

**Fig. 5:**
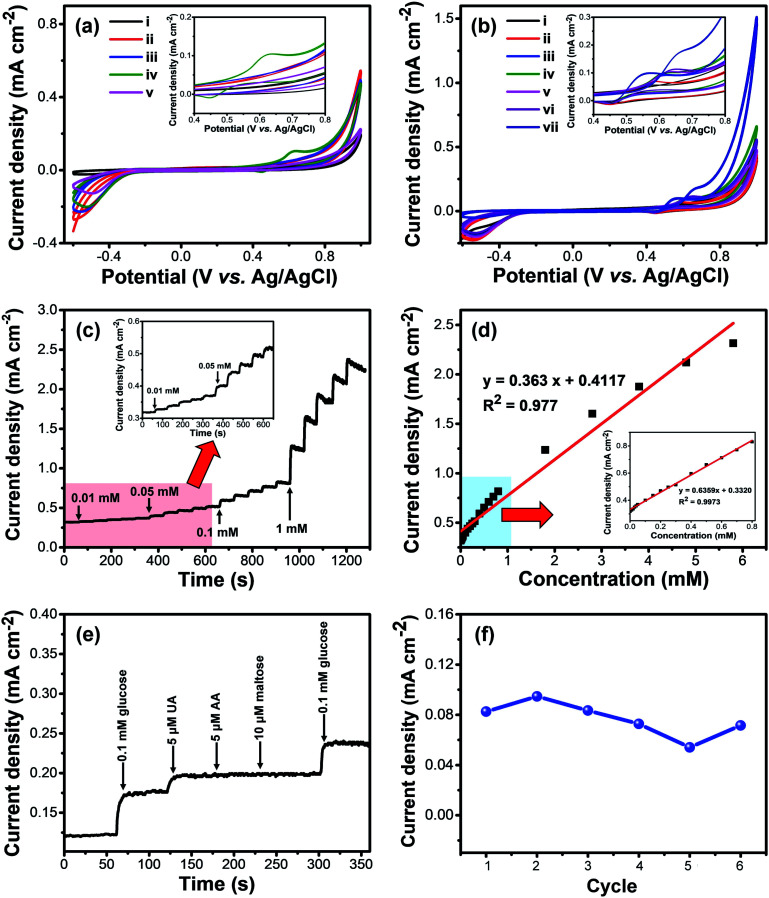
(a) CV curves of bare GCE (i) and GCEs coated with hierarchical sheet-like M-BDC toward 5 mM of glucose in 0.1 M NaOH: (ii) Cu-BDC, (iii) Mn-BDC, (iv) Ni-BDC, and (v) Zr-BDC. (b) CV curves of hierarchical sheet-like Ni-BDC electrode to different concentrations of glucose in 0.1 M NaOH: (i) 0 mM (without glucose), (ii) 0.1 mM, (iii) 1 mM, (iv) 2 mM, (v) 5 mM, (vi) 10 mM, and (vii) 20 mM. (c) Amperometric (*i*–*t*) curves of the hierarchical sheet-like Ni-BDC electrode with successive additions of glucose in 0.1 M NaOH at 0.63 V (*versus* Ag/AgCl). (d) The changes in current density of the hierarchical sheet-like Ni-BDC electrode with increasing glucose concentration. (e) Amperometric responses of the hierarchical sheet-like Ni-BDC electrode upon addition of glucose (0.1 mM) and various interfering compounds (5 μM) in 0.1 M NaOH. (f) Variation of the current response to 0.1 mM glucose for the hierarchical sheet-like Ni-BDC electrode for 6 consecutive cycles after 50 days of storage time.

Time-dependent experiments were performed to study the growth mechanisms of M-BDC MOFs (Fig. 3). For Cu-BDC, square-like plate particles are readily observed within 2 h (Fig. 3a-1) and the stacking of these square plate-like particles is intensified with the increase of reaction time from 4 to 24 h (Fig. 3a-2 to Fig. 3a-5), leading to the formation of hierarchical plate-like Cu-BDC particles. For Mn-BDC, microspindles are observed after 2 h (Fig. 3b-1) and 4 h (Fig. 3b-2) and slowly, plate-like particles are growing from these microspindles after 8 h, as seen in Fig. 3b-3. After 16 h, more plate-like particles are formed on these large spindle-like particles (Fig. 3b-4). Eventually, hierarchical plate-like Mn-BDC particles are achieved after 24 h of reaction (Fig. 3b-5). For Ni-BDC, bulk particles with irregular structure are obtained after 2 h (Fig. 3c-1). After 4 h, these bulk particles begin to self-organize into aggregated sheet-like particles (Fig. 3c-2) and this self-assembly process continues between 8–16 h (Fig. 3c-3 and c-4). As seen in Fig. 3c-5, well-defined hierarchical multilayered sheet-like Ni-BDC particles are successfully formed after 24 h. For Zr-BDC, the product obtained after 2 h consists mostly of aggregated bulk particles (Fig. 3d-1). After 4 h, these bulk particles separate into smaller aggregated nanoparticles with irregular structure (Fig. 3d-2). After 8 h, these aggregated nanoparticles become increasingly separated from each other (Fig. 3d-3) and slowly grow into plate-like particles after 16 h (Fig. 3d-4). Finally, uniform plate-like Zr-BDC particles are achieved after 24 h of reaction (Fig. 3d-5).

The effects of the reaction temperature on the formation of M-BDC MOFs in the absence of PVP are shown in Fig. S1.† With the exception of Cu-BDC, all the other M-BDC MOFs cannot be formed at room temperature. Cu-BDC generates thin, highly interconnected square-like sheets with an average length of 2 μm at room temperature. Upon heating to higher temperatures between 55–95 °C, the square sheet-like particles become more well-separated without a significant change in particle size. However, when the temperature is raised to 115 °C, these sheets become stacked on top of each other. This stacking effect is further intensified at 135 °C. In contrast, Ni-BDC starts to precipitate at 75 °C, showing irregular plate-like structures that assemble into 2D stacked structures at higher temperatures (*i.e.*, 115 °C and 135 °C). Mn-BDC precipitates at 95 °C and the product displays an aggregated bundle-like structure, and the increase of reaction temperature to 115 °C leads to the separation of these bundles into individual nanorods and eventually, thick hierarchical microrods are achieved at 135 °C. In comparison, Zr-BDC gradually transforms from agglomerated nanoparticles at 95 °C to hierarchical spheres composed of small nanoparticles at 115 °C and ultimately to hierarchical plate-like networks at 135 °C. Based on these results, it is evident that the modification of reaction temperature alone is generally not sufficient for achieving the hierarchical 3D M-BDC MOFs. Therefore, in this work, we have employed PVP to promote the formation and growth of hierarchical sheet/plate-like M-BDC MOFs.

PVP is frequently used as a surfactant and shape directing agent in nanoparticle synthesis methods.^39^ The pyrrolidone moiety of PVP has a highly polar amide group that reversibly interacts with polarizable ions and charged molecules and has been used to synthesize MOFs.^41^ The concentration of PVP is also important for creating the hierarchical sheet/plate-like structures because PVP can generate depletion forces between larger particles including nanosheets, driving them to coagulate and self-assemble.^42,43^ To identify the role of PVP, a series of control experiments were carried out by modifying the mass ratio of metal precursor to PVP (*i.e.*, 1 : 1, 1 : 3, and 1 : 5) during the synthesis of each M-BDC MOF. Thick square plate-like Cu-BDC particles are obtained at a Cu precursor/PVP mass ratio of 1 : 1 (Fig. S2a†), whereas hierarchical square plate-like particles are achieved at an optimal ratio of 1 : 3 (Fig. 1a, b and S2b†). A further increase of the Cu precursor/PVP mass ratio to 1 : 5 yields aggregated nanoparticles as the product (Fig. S2c†). In comparison, the Mn-BDC product obtained at a Mn precursor/PVP mass ratio of 1 : 1 displays bulk dumbbell-like morphology (Fig. S2d†). The increase of the Mn precursor/PVP mass ratio to 1 : 3 generates oval-like particles with an average size of 2 μm (Fig. S2e†). Finally, Mn-BDC particles with uniform sheet-like morphology are achieved at an optimized Mn precursor/PVP mass ratio of 1 : 5 (Fig. 1c, d and S2f†).

For Ni-BDC, stacked square-like particles are observed at a Ni precursor/PVP mass ratio of 1 : 1 (Fig. S2g†), which are transformed into hierarchical sheet-like particles with the increase of the Ni precursor/PVP mass ratio to 1 : 3 (Fig. 1g, h and S2h†). In contrast, the Ni-BDC product achieved at a higher Ni precursor/PVP mass ratio of 1 : 5 exhibits a net-like structure with many holes, as seen in Fig. S2i.† For Zr-BDC, hierarchical plate-like particles are readily observed at a Zr precursor/PVP mass ratio of 1 : 1 (Fig. S2j†), and the diameters of these plates are enlarged with a further increase of the Zr precursor/PVP mass ratio to 1 : 3 (Fig. S2k†). Eventually, a hierarchical structure assembled from well-separated plates is achieved at an optimized Zr precursor/PVP mass ratio of 1 : 5 (Fig. 1e, f and S2l†). In general, we can conclude that PVP facilitates the growth of hierarchical sheet/plate-like M-BDC, but excess PVP in the M-BDC growth solution leads to excessive stacking of the nanosheets or nanoplates to form bulk irregular crystals due to depletion attraction.

The third set of control experiments involves the removal of acetonitrile from the growth solution of M-BDC with the metal precursor to PVP mass ratio fixed at the optimized ratio for each M-BDC sample. In the absence of acetonitrile, none of the M-BDC samples exhibit hierarchical sheet/plate-like morphology. The Cu-BDC product achieved in the absence of acetonitrile consists of stacked square-like particles with sizes between 500 nm to 2.5 μm (Fig. S3a†), whereas the Mn-BDC and Ni-BDC products consist of bulk crystals (Fig. S3b and c†). In contrast, the Zr-BDC sample obtained without acetonitrile is made up of aggregated quasi-cubic-like particles (Fig. S3d†). The above findings highlight the important role of acetonitrile as the removal of acetonitrile causes poor solvation of the metal ions, which therefore interrupts the formation of hierarchical sheet/plate-like M-BDC MOFs.

The overall formation mechanism is proposed as follows. Acetonitrile serves primarily to improve the solvation of metal ions in solution.^44^ DMF begins to decompose at ∼130 °C in the presence of acid, generating carbon monoxide and dimethylamine molecules. The dimethylamine molecules have a relatively high p*K*_a_ and deprotonate additional H_2_BDC linker molecules that may bond with metal precursors to form the MOF crystal. PVP plays an essential role in crystallization because it reduces the rate of crystal growth by forming weak hydrogen bonds with organic molecules,^45^ and stronger <svg xmlns="http://www.w3.org/2000/svg" version="1.0" width="10.400000pt" height="16.000000pt" viewBox="0 0 10.400000 16.000000" preserveAspectRatio="xMidYMid meet"><metadata> Created by potrace 1.16, written by Peter Selinger 2001-2019 </metadata><g transform="translate(1.000000,15.000000) scale(0.011667,-0.011667)" fill="currentColor" stroke="none"><path d="M80 1160 l0 -40 40 0 40 0 0 -40 0 -40 40 0 40 0 0 -40 0 -40 40 0 40 0 0 -40 0 -40 40 0 40 0 0 -40 0 -40 40 0 40 0 0 -40 0 -40 40 0 40 0 0 -40 0 -40 40 0 40 0 0 80 0 80 -40 0 -40 0 0 40 0 40 -40 0 -40 0 0 40 0 40 -40 0 -40 0 0 40 0 40 -40 0 -40 0 0 40 0 40 -40 0 -40 0 0 40 0 40 -80 0 -80 0 0 -40z M560 520 l0 -40 -40 0 -40 0 0 -40 0 -40 -40 0 -40 0 0 -40 0 -40 -40 0 -40 0 0 -40 0 -40 -40 0 -40 0 0 -40 0 -40 -40 0 -40 0 0 -40 0 -40 -40 0 -40 0 0 -40 0 -40 80 0 80 0 0 40 0 40 40 0 40 0 0 40 0 40 40 0 40 0 0 40 0 40 40 0 40 0 0 40 0 40 40 0 40 0 0 40 0 40 40 0 40 0 0 80 0 80 -40 0 -40 0 0 -40z"/></g></svg> C=O→M bonds with metal cations and metal surfaces.^46,47^ Therefore, PVP initially serves as a dynamic hydrophobic/hydrophilic environment enabling reversible interactions to reduce the ionic mobility of the metal precursors and promote nucleation of MOF nanocrystals. During the growth phase, PVP tends to bond more strongly to one facet or more facets of the MOF crystal. Preferential binding promotes shape-control, which in the case of M-BDC generates hierarchical nanosheets/nanoplates depending on the metal. Shape-control is lost when only small amounts of PVP are used, or acetonitrile is omitted (Fig. S2 and S3†), resulting in bulk crystals. PVP also plays a secondary role in assisting the formation of the hierarchical structures as mentioned above.

N_2_ adsorption–desorption measurements were used to characterize the specific surface area and pore size distribution of the as-prepared M-BDC samples. Fig. 4a, b, and Table S2† reveal that the trend in specific surface area is in the order of Zr-BDC > Mn-BDC > Cu-BDC > Ni-BDC. Our M-BDC samples exhibit lower surface areas than some previously reported 2D MOFs, but this is likely due to the presence of PVP, which can lead to partial blocking of the internal space of the M-BDC crystals.^48^ Nonetheless, BDC-based MOFs with surface areas lower than 100 m^2^ g^−1^ have been reported previously.^49–51^ The pore volume of the as-synthesized Zr-BDC, Mn-BDC, Cu-BDC, and Ni-BDC samples are 1.914, 0.566, 0.308, and 0.207 cm^3^ g^−1^, respectively. The pore size distribution curves of the hierarchical sheet/plate-like M-BDC MOFs were calculated using the non-local density functional theory (NLDFT) method. The as-prepared Cu-BDC, Mn-BDC, and Ni-BDC samples are mesoporous with mesopore peaks at 26.4, 26.3, and 13.7 nm, as shown in Fig. 4c–e, respectively. In contrast, Zr-BDC has a much higher surface area compared to the other M-BDC MOFs due to its microporous nature with a main peak at 2.67 nm (Fig. 4f).

### 3.2. Non-enzymatic glucose sensing performance

The hierarchical structure and exposed metal sites of the M-BDC architectures make them attractive for sensing applications. The as-prepared M-BDC samples were coated onto glassy carbon electrodes (GCEs) and used for electrochemical non-enzymatic glucose sensing. The glucose sensing measurements were carried out by using an electrochemical workstation with a three-electrode system. Fig. 5a displays the electrochemical responses of the hierarchical sheet/plate-like M-BDC electrodes to 5 mM of glucose in 0.1 M NaOH electrolyte in the potential range of −0.6 to 1.0 V. Among all the samples, only Ni-BDC shows a pair of asymmetric redox peaks in the CV curves with an anodic peak at around 0.63 V and a cathodic peak at 0.45 V, indicating the presence of redox reactions between Ni^2+^/Ni^3+^ and OH^−^ with reversible faradaic mechanism to form NiOOH and Ni(OH)_2_.^52–54^ Although other M-BDC electrodes do not show any asymmetric redox peaks, the current densities of GCEs coated with the M-BDC samples are still much higher than that of bare GCE, as seen in Fig. 5a. Nonetheless, as only the Ni-BDC electrode displays a significant response to glucose, subsequent glucose sensing measurements will focus solely on this MOF.

The electrochemical glucose sensing performance of the hierarchical sheet-like Ni-BDC was compared with that of bulk Ni-BDC. The bulk Ni-BDC (Fig. S3c†) was synthesized with a Ni precursor/PVP ratio of 1 : 3 at 135 °C without acetonitrile. Compared to the hierarchical sheet-like Ni-BDC, the asymmetric redox peaks of bulk Ni-BDC show potential shift to the negative direction (0.64 V to 0.56 V) with increasing glucose concentration from 5 to 20 mM (Fig. S4†). However, the current density remains more or less similar at ∼0.13 mA cm^−2^. In comparison, the current density of the hierarchical sheet-like Ni-BDC ranges from ∼0.14 to 0.177 mA cm^−2^ with a potential shift to the positive direction (from 0.63 V to 0.67 V) using the same glucose concentration range (Fig. 5b). These results indicate the superior glucose sensing performance of the hierarchical sheet-like Ni-BDC compared to the bulk Ni-BDC, owing to the more accessible and increased active sites as well as the interconnected 3D structure which can provide interspaces for the diffusion of biomolecules, decrease the contact resistance, and enhance the mass or charge transfer rate at the interface between the electrode and the electrolyte.^55^

**Scheme 1:**
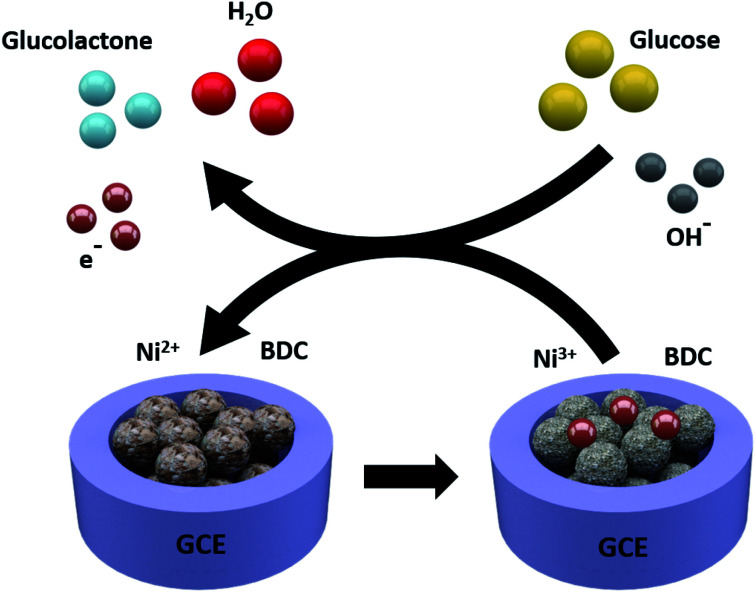
A schematic illustration depicting the electrochemical glucose sensing mechanism of the hierarchical sheet-like Ni-BDC sensor.

The responses of hierarchical sheet-like Ni-BDC electrode to various concentrations of glucose (0.1, 1.0, 2.0, 5.0, 10.0 and 20.0 mM) were investigated by cyclic voltammetry (CV) (Fig. 5c). The current density of the Ni-BDC electrode increases with increasing glucose concentration, accompanied by a positive potential shift. These observations suggest that Ni^2+^ and OH^−^ redox reactions are involved. The electrochemical glucose sensing mechanism of the hierarchical sheet-like Ni-BDC electrode is based on the possible redox reactions of Ni^2+^ with glucose in the NaOH electrolyte (Scheme 1) as expressed by the following equations:^53,56,57^

1 Ni^2+^–H_2_BDC → Ni^3+^–H_2_BDC + e^−^

2 Ni^3+^–H_2_BDC + OH^−^ + glucose → Ni^2+^–H_2_BDC glucolactone + H_2_O + e^−^

The sensitivity and the response time of the hierarchical sheet-like Ni-BDC electrode toward different concentrations of glucose were determined by amperometric measurements. Fig. 5c displays the amperometric responses of Ni-BDC upon additions of varying concentrations of glucose at 0.63 V. It is evident that the current density of the hierarchical sheet-like Ni-BDC electrode gradually increases with increasing glucose concentration. The response time was measured from the initial time at which the glucose solution was added to the electrolyte solution until the current density became stable. The response times of the hierarchical sheet-like Ni-BDC electrode to 0.01, 0.05, 0.1, and 1 mM of glucose are 3, 3, 4, and 5 s, respectively, indicating the fast response time of the hierarchical sheet-like Ni-BDC sensor. From Fig. 5c, we can plot the glucose concentration *versus* the current density curve to obtain the electrode calibration curve, as shown in Fig. 5d. Evidently, there is a linear correlation between the glucose concentration and the current density in the range of 0.01 mM to 0.8 mM with a correlation coefficient of 0.9973. The hierarchical sheet-like Ni-BDC sensor exhibits a relatively high sensitivity of 635.9 μA mM^−1^ cm^−2^. The limit of detection (LoD) for glucose is determined to be 6.68 μM (S/N = 3).

Table 1 compares the electrochemical glucose sensing performance of various non-enzymatic catalysts with the as-prepared hierarchical sheet-like Ni-BDC sensor.^58–68^ It can be observed from Table 1 that the hierarchical sheet-like Ni-BDC electrode shows higher sensitivity than non-enzymatic glucose sensors based on Ni(OH)_2_/Au,^60^ Ni(OH)_2_ nanoparticles/reduced graphene oxide (RGO),^61^ GO_*x*_/*p*-NiO/*n*-Bi_4_Ti_3_O_12_,^66^ NiCo layered double hydroxide (LDH) nanosheets/graphene nanoribbons,^67^ and even some noble metal composites, such as Pt@carbon nano-onions,^64^ Ni–Pd@activated carbon,^65^ and PtPd/porous holey nitrogen-doped graphene.^68^ Zhang *et al.*^33^ previously compared the glucose sensing performance of bulk Ni-BDC with that of Ni-BDC/carbon nanotube (CNT) hybrid. They found that the bulk Ni-BDC did not show redox peaks during CV scans. However, the response was significantly increased when the Ni-BDC MOF was mixed with CNTs. In comparison, the as-synthesized hierarchical sheet-like Ni-BDC sensor shows a higher sensitivity to glucose than this Ni-BDC/CNT hybrid even without the addition of carbon-based materials or the use of conductive nickel foam. Furthermore, compared with hierarchical flower-like Ni-BDC-SWCNT (single-wall carbon nanotubes)/GCE,^58^ our hierarchical sheet-like Ni-BDC sensor exhibits superior sensitivity to glucose even without the addition of SWCNT. This indicates that the catalytic activity of Ni-BDC may be enhanced by tuning the morphology to a hierarchical 3D structure composed of 2D nanoarchitectures. Therefore, the good electrochemical glucose sensing performance of the Ni-BDC is largely attributed to its nanosheet-assembled hierarchical 3D structure, which can provide open pores and numerous accessible redox sites that are accessible due to the interconnected nature of the nanosheets and nanoplates.

**Table 1:** Comparison of the electrochemical glucose sensing performance of the as-prepared hierarchical sheet-like Ni-BDC sensor with previously reported non-enzymatic glucose sensors^a^

Electrode composition	Sensitivity (μA mM^−1^ cm^−2^)	Linear range (mM)	LoD (μM)	Reference
Hierarchical flower-like Ni-BDC-SWCNT/GCE	N/A	0.02–4.4	4.60	58
NiO/Ni foam	395	0.018–1.2	6.15	59
Ni(OH)_2_/Au	371	0.005–2.2	0.92	60
Ni(OH)_2_ NPs/RGO/GCE	11.4	0.002–3.1	0.60	61
α-Ni(OH)_2_/FTO	446	0.01–0.75	3.00	62
Iridium(iii)-MOFs	—	0.05–5.0	10.0	63
Pt@carbon nano-onions	21.6	2–28	90	64
Ni–Pd@AC/GCE	90	0.01–1	0.014	65
GO_*x*_/*p*-NiO/*n*-Bi_4_Ti_3_O_12_	215	0.02–3.55	1.26	66
NiCo LDH nanosheets/graphene nanoribbons	344	0.005–0.8	0.6	67
PtPd/porous holey nitrogen-doped graphene	52.5	0.1–4	1.82	68
Hierarchical sheet-like Ni-BDC/GCE	636	0.01–0.8	6.68	This work

a BDD: boron-doped diamond; NP: nanoparticles; btc: benzene-1,3,5-tricarboxylic acid; SWCNT: single-walled carbon nanotubes; AC: activated carbon; GCE: glassy carbon electrode; FTO: fluorine-doped tin oxide; LDH: layered double hydroxide.

In clinical use, an electrochemical sensor must be able to distinguish between the target molecule and the interfering molecules in the sample test (*e.g.*, blood, urine, saliva, *etc.*), especially in the case of non-enzymatic sensors. Therefore, the selectivity of the hierarchical sheet-like Ni-BDC electrode toward glucose was checked against other common interferents found in blood, such as uric acid (UA), ascorbic acid (AA), and maltose, as shown in Fig. 5e. The amperometric responses of Ni-BDC to 0.1 mM of glucose, 5 μM of UA, 5 μM of AA, and 10 μM of maltose in 0.1 M NaOH at 0.63 V are largely different. Changes in current density of 50.99 and 19.8 μA cm^−2^ are observed after additions of glucose and UA into the electrolyte, respectively. If the response of the hierarchical sheet-like Ni-BDC to UA is compared with that to glucose, Ni-BDC has 100% selectively to glucose compared to only 38.88% for UA, implying that these two molecules can still be distinguished. In contrast, no significant changes in current density are observed after additions of AA and maltose, indicating the good selectivity of the hierarchical sheet-like Ni-BDC electrode toward glucose. The stability of the Ni-BDC electrode was examined by measuring its current response toward 0.1 mM glucose for 6 consecutive cycles after prolonged storing (50 days). As shown in Fig. 5f, the hierarchical sheet-like Ni-BDC electrode can retain 88% of its original value after 6 consecutive sensing cycles, even after prolonged storing, thus indicating its relatively stable sensing performance. The SEM images of the Ni-BDC electrode before and after this stability test (Fig. S5a and b†) reveal that the sheet-like structure is still maintained, however they become more crumpled in appearance. To further assess the stability of the as-prepared Ni-BDC electrode, we have carried out a leaching test on the glucose-containing NaOH solution after the sensing measurement to identify whether Ni metal has leaked into the solution. The atomic absorption spectroscopy (AAS) measurement reveals that the concentration of Ni metal in this solution is negligible (−0.0042), indicating that the Ni metal in the Ni-BDC electrode has not been leaked into the solution. The XRD analysis of the Ni-BDC electrode after the glucose sensing test shows that the Ni-BDC becomes more amorphous. However, several weak peaks belonging to Ni-BDC can still be observed after the sensing test, as indicated by the circles on Fig. S5c,† indicating its respectable stability.

## 4. Conclusions

In summary, this work describes a general route to synthesize hierarchical 3D M-BDC (M = Cu, Mn, Ni, and Zr) MOFs which are assembled from two-dimensional nanosheets/nanoplates in the presence of PVP and acetonitrile as shape-directing agents. The mass ratio of the metal precursor to PVP and the amount of acetonitrile strongly influence the formation of the hierarchical sheet/plate-like M-BDC MOFs. Acetonitrile helps maintain the solvation of metal precursor, while PVP assists in the nucleation and growth of the MOF crystals. Removal of either acetonitrile or PVP results in bulk MOF crystals. When employed for non-enzymatic electrochemical glucose sensing, only the hierarchical sheet-like Ni-BDC electrode shows a significant amperometric response toward glucose with a high sensitivity of 635.9 μA mM^−1^ cm^−2^ with a wide linear range between 0.01 to 0.8 mM. The limit of detection (LoD) of the hierarchical sheet-like Ni-BDC electrode toward glucose is around 6.68 μM (S/N = 3). It is expected that this work will provide useful strategies for future synthesis of hierarchical 3D MOFs and promote the direct utilization of MOFs in other electrochemical applications. In addition, these MOFs can be utilized in the future as precursors for creating hierarchical metal oxides, carbons, and their hybrid materials.^14,69,70^

## Conflicts of interest

There are no conflicts to declare.

## Supplementary Material

SC-011-C9SC05636J-s001
