# Supplementary material for: General synthesis of hierarchical sheet/plate-like M-BDC (M = Cu, Mn, Ni, and Zr) metal–organic frameworks for electrochemical non-enzymatic glucose sensing
Source: Chem Sci. 2020 Mar 12;11(14):3644–55. doi: 10.1039/c9sc05636j (PMC8152586; doi:10.1039/c9sc05636j)
Supplement: SC-011-C9SC05636J-s001 [file SC-011-C9SC05636J-s001.pdf]

Electronic Supplementary Material (ESI) for Chemical Science.
This journal is © The Royal Society of Chemistry 2020

Table S1. Refined unit cell parameters for as-prepared M-BDC MOFs

MOF	R_p	R_{wp}	a	b	c	α	β	γ
Cu-BDC	11.261	19.314	11.324215	14.325037	7.782220	90.000	108.269	90.000
Mn-BDC	7.967	116.414	24.792263	10.585683	17.421875	90.000	130.017	90.000
Zr-BDC	8.768	117.944	20.742634	20.742634	20.742634	90.000	90.000	90.000
Ni-BDC	18.987	65.520	12.987082	11.387417	17.896505	90.000	96.718	90.000

Table S2. Specific surface area and pore size distribution of the hierarchical M-BDC (M = Cu, Mn, Ni, and Zr).

Sample	Surface area (m^2 g^{-1})	Pore volume (cm^3 g^{-1})	Pore width (nm)
Cu-BDC	90.2	0.308	26.384
Mn-BDC	93.7	0.566	26.384
Ni-BDC	34.7	0.207	13.709
Zr-BDC	1248.4	1.914	2.657

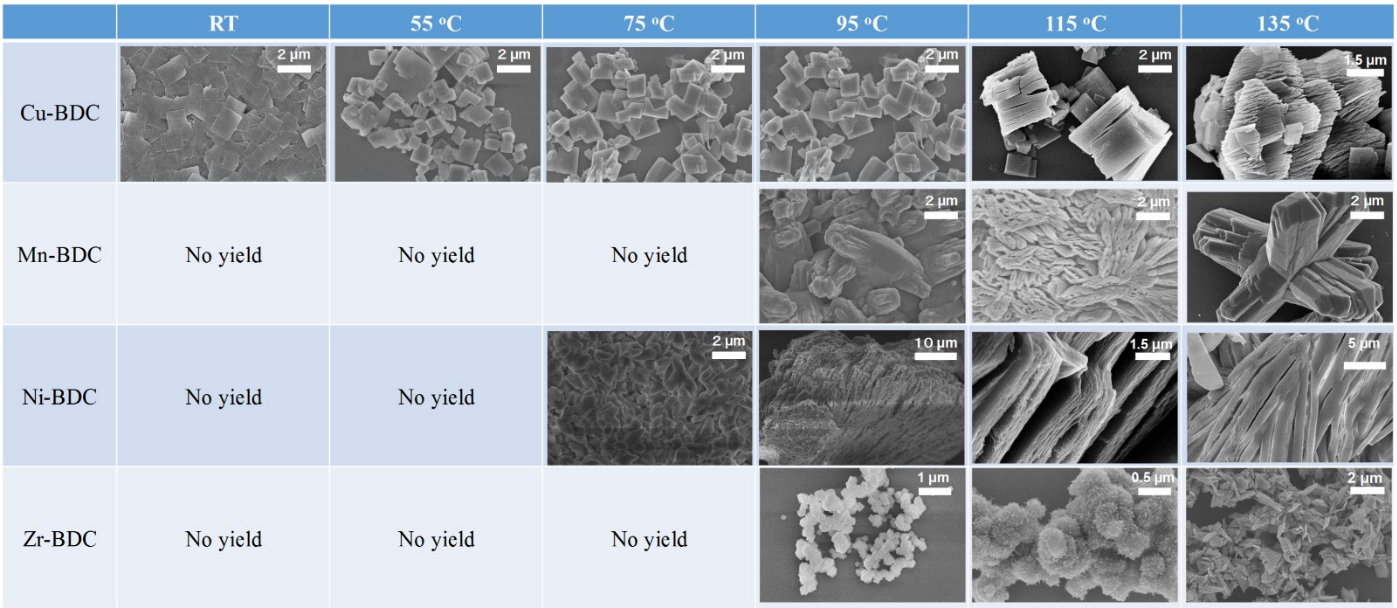

Fig. S1. SEM images of M-BDC (M= Cu, Mn, Ni, and Zr) samples obtained at RT, 55 °C, 75 °C, 95 °C, 115 °C, and 135 °C in the absence of PVP.

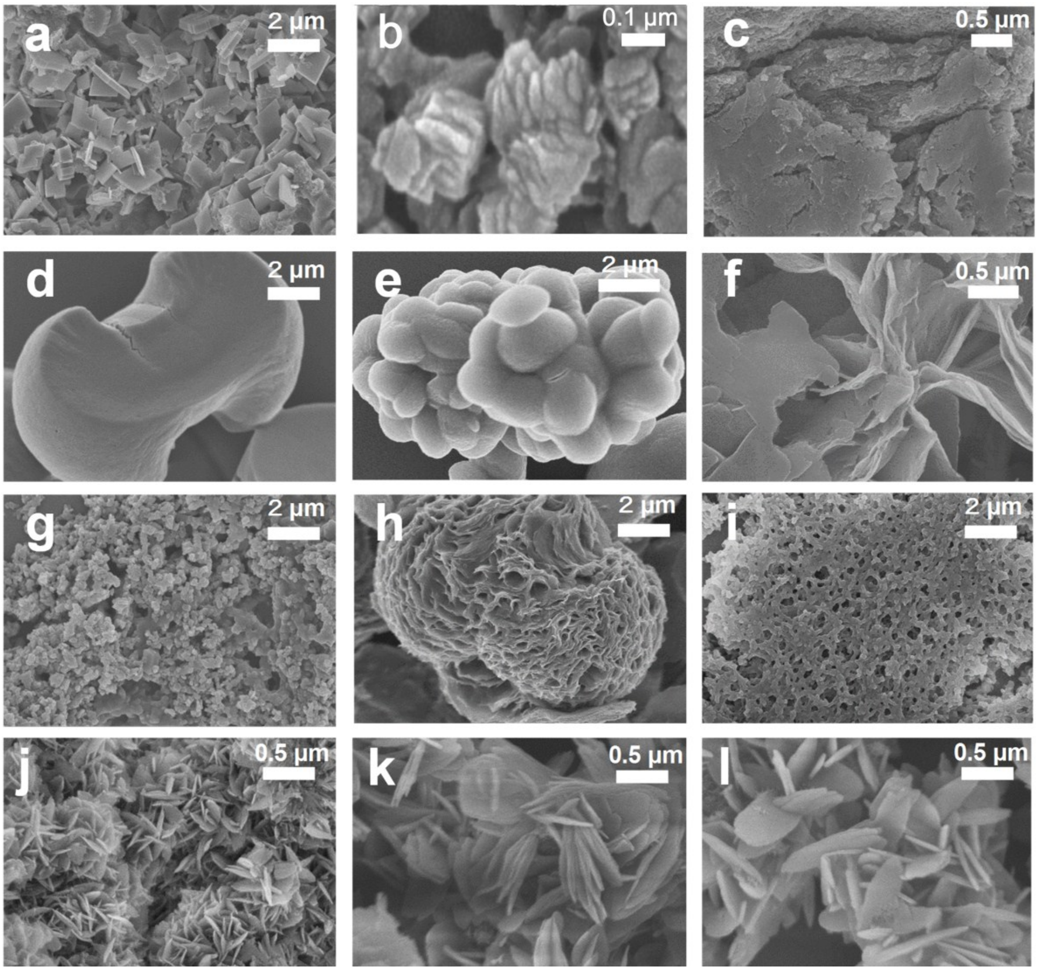

Fig. S2. SEM images of Cu-BDC samples obtained with copper precursor: PVP ratios of (a) 1:1, (b) 1:3, and (c) 1:5 at 135 °C. SEM images of Mn-BDC samples obtained with manganese precursor: PVP ratios of (d) 1:1, (e) 1:3, and (f) 1:5. SEM images of Ni-BDC samples obtained with nickel precursor: PVP ratios of (g)

1:1, (h) 1:3, and (i) 1:5. SEM images of Zr-BDC samples obtained with zirconium precursor: PVP ratios of (j) 1:1, (k) 1:3, and (l) 1:5.

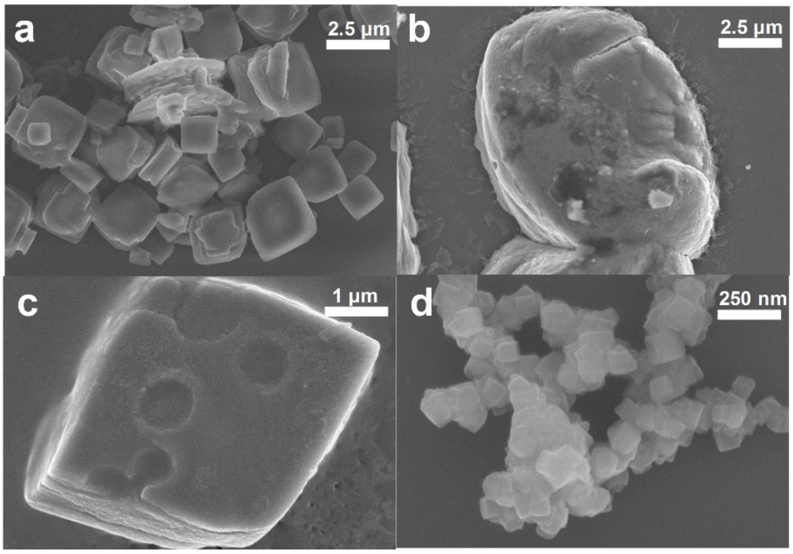

Fig. S3. SEM images of M-BDC samples synthesized with the optimized mass ratios of metal precursor to PVP without acetonitrile: (a) Cu-BDC (1:3), (b) Mn-BDC (1:5), (c) Ni-BDC (1:3), and (d) Zr-BDC (1:5).

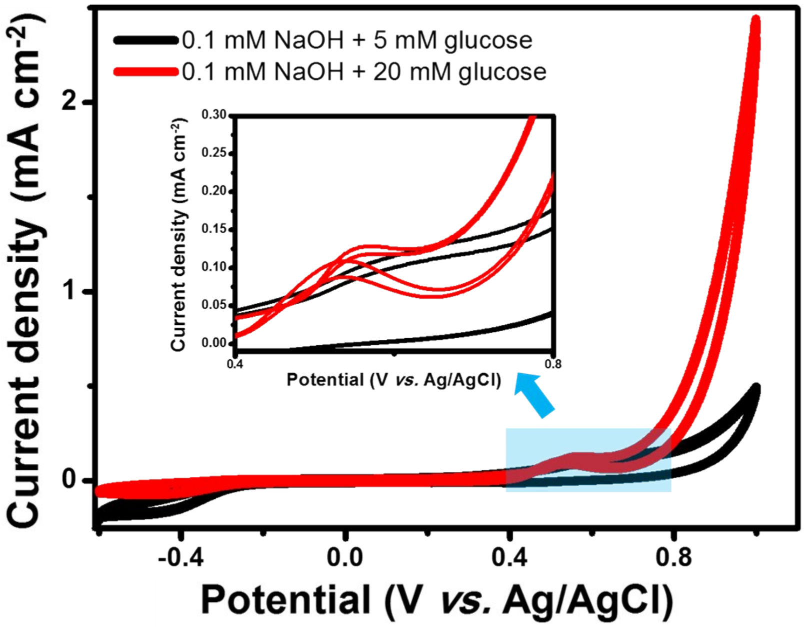

Fig. S4. CV curves of bulk Ni-BDC in 0.1 M NaOH in the presence of 5 mM and 20 mM of glucose.

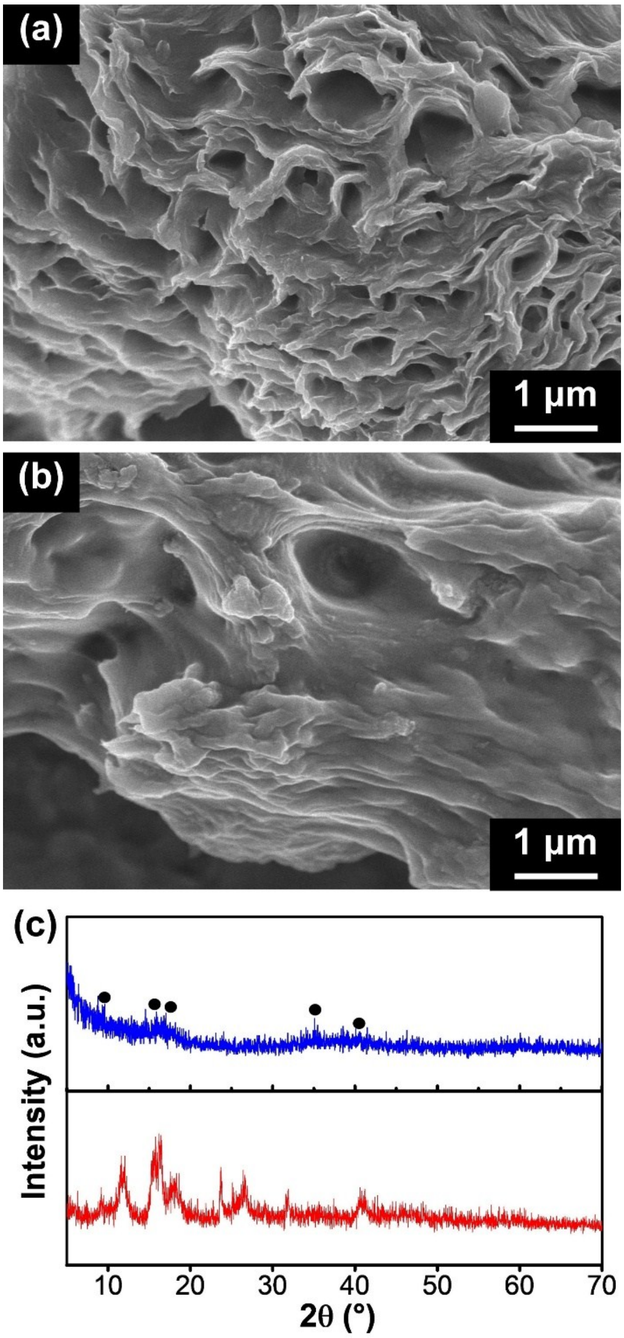

Fig. S5. SEM images of the hierarchical sheet-like Ni-BDC before (a) and after the stability test (b). (c) XRD patterns of the hierarchical sheet-like Ni-BDC before (i) and after the sensing test (ii).
